# Evaluating a new generation of wearable high-density diffuse optical tomography technology via retinotopic mapping of the adult visual cortex

**DOI:** 10.1117/1.NPh.8.2.025002

**Published:** 2021-04-09

**Authors:** Ernesto E. Vidal-Rosas, Hubin Zhao, Reuben W. Nixon-Hill, Greg Smith, Luke Dunne, Samuel Powell, Robert J. Cooper, Nicholas L. Everdell

**Affiliations:** a University College London, Diffuse Optical Tomography of the Human Brain Research Group, Biomedical Optics Research Laboratory, Department of Medical Physics and Biomedical Engineering, London, United Kingdom; b University of Glasgow, James Watt School of Engineering, Glasgow, United Kingdom; c Imperial College London, Department of Mathematics, London, United Kingdom; d Gowerlabs Ltd., London, United Kingdom; e Nottingham University, Department of Electrical and Electronic Engineering, Nottingham, United Kingdom

**Keywords:** high-density diffuse optical tomography, functional near-infrared spectroscopy, wearable, short-separation regression, visual stimuli

## Abstract

**Significance:** High-density diffuse optical tomography (HD-DOT) has been shown to approach the resolution and localization accuracy of blood oxygen level dependent-functional magnetic resonance imaging in the adult brain by exploiting densely spaced, overlapping samples of the probed tissue volume, but the technique has to date required large and cumbersome optical fiber arrays.

**Aim**: To evaluate a wearable HD-DOT system that provides a comparable sampling density to large, fiber-based HD-DOT systems, but with vastly improved ergonomics.

**Approach**: We investigated the performance of this system by replicating a series of classic visual stimulation paradigms, carried out in one highly sampled participant during 15 sessions to assess imaging performance and repeatability.

**Results**: Hemodynamic response functions and cortical activation maps replicate the results obtained with larger fiber-based systems. Our results demonstrate focal activations in both oxyhemoglobin and deoxyhemoglobin with a high degree of repeatability observed across all sessions. A comparison with a simulated low-density array explicitly demonstrates the improvements in spatial localization, resolution, repeatability, and image contrast that can be obtained with this high-density technology.

**Conclusions**: The system offers the possibility for minimally constrained, spatially resolved functional imaging of the human brain in almost any environment and holds particular promise in enabling neuroscience applications outside of the laboratory setting. It also opens up new opportunities to investigate populations unsuited to traditional imaging technologies.

## 1 Introduction

The investigation of human brain function has made dramatic progress since the introduction and development of functional neuroimaging. Functional magnetic resonance imaging (fMRI) and positron emission tomography (PET) have been at the forefront of this development, but they are not free of limitations. Both impose significant constraints on the mobility of participants, which hinders their application in challenging populations such as infants and in the study of neural processes and behaviors that involve movement. Extended or repeated monitoring is also difficult due to the associated costs, the confined scanner environment and (in the case of PET) the use of radiotracers.^1^^,^^2^ In addition, fMRI has contraindications for electronic or metal implants (such as pacemakers, cochlear implants, aneurysm clips, and surgical devices). Owing to the large, fixed nature of MRI and PET equipment, and the requirement on the participant to lie flat, it is very difficult to study the brain in everyday scenarios, such as during face-to-face conversations.

Diffuse optical methods have shown great promise in overcoming some of these limitations in recent years.^3^^,^^4^ Functional near-infrared spectroscopy (fNIRS) uses near-infrared light to interrogate cerebral function. It employs an array of optical sources and detectors placed on the scalp to monitor the changes in cerebral oxyhemoglobin and deoxyhemoglobin concentration and can provide two-dimensional images with a spatial resolution on the order of 3 cm.^5^^,^^6^ High-density diffuse optical tomography (HD-DOT) is an extrapolation of fNIRS methodology that employs a high-density measurement array. Although the definition of “high density” in this context has not been precisely established, an appropriate definition would be that a HD-DOT array provides channels with several different source–detector separations spanning the “short separation (SS)” (<15  mm) to “long” (≥30  mm) range and provides overlapping spatial sensitivity profiles at each of these separations throughout the field of view. It is now well established that HD-DOT can provide depth-resolved images of superior quality to fNIRS or other diffuse optical imaging approaches.^7^^–^^9^ The mutual information obtained from the plurality of overlapping channel measurements increases spatial resolution, and the use of multiple source–detector separations improves both lateral and depth specificity. Furthermore, sampling at different source–detector separations provides a means to reduce the influence of signals from extracerebral tissues.^10^^,^^11^

Zeff et al.^7^ first demonstrated the potential of HD-DOT by monitoring functional brain activation due to visual stimuli. Using a similar paradigm, Eggebrecht et al.^9^ produced cortical retinotopic maps of the visual field and demonstrated that HD-DOT approaches the resolution and location accuracy of fMRI at the level of the cortex. Using a motor paradigm, Habermehl et al.,^12^ resolved the somatotopy mapping of two fingers with a commercially available instrument (DYNOT, NIRx Medizintechnik GmbH, Berlin, Germany). HD-DOT has also been used to analyze language processing^13^ and resting-state functional connectivity in adults^14^ and neonates,^15^ with results that are largely consistent with prior fMRI research. Earlier this year, Fishell et al.^16^ conducted cognitive experiments on children living in a resource-limited environment using a portable, fiber-based HD-DOT system.

The introduction of HD-DOT has moved forward the limits of diffuse optical approaches, but there is still potential for significant improvement. It has been demonstrated in recent years that fNIRS devices can be built to be compact enough to be wearable, which opens up the possibility of studying the brain in novel situations and using paradigms that are beyond what is possible in the laboratory and clinical settings.^17^^,^^18^ This generation of wearable fNIRS devices has allowed participants to be monitored as they move freely, and in naturalistic environments. For example, fNIRS has been used in healthy adults to study the cognitive load in dual-task cognitive-motor protocols. The primary task is usually walking, whereas the second activity has included a broad range of tasks including performing mathematical operations,^19^^,^^20^ attention-demanding tasks,^21^ and memory tasks.^22^^,^^23^ Wearable fNIRS has also been used in patients with neurodegenerative disorders and cognitive disabilities, for instance, mild cognitive impairment^24^ and Parkinson’s disease.^25^ Other examples of wearable fNIRS experimentation include monitoring the brain during table tennis,^26^ outdoor cycling,^27^ unconstrained street exploration,^28^ and even while actors perform in a play.^29^ There is also growing interest in exploring brain function in other scenarios, including the study of the social brain during real-world interactions using hyperscanning approaches,^30^^,^^31^ for brain–computer interfaces, and neuroeducation.^32^ The signal conditioning necessary to undertake these studies, such as the removal of movement artifacts or the reduction of systemic signals, is under intensive research.^33^

Although these applications show what is now achievable with wearable fNIRS, existing devices maintain many of the same challenges as traditional, fiber-based fNIRS systems: poor resolution, a lack of depth specificity (and therefore a vulnerability to superficial hemodynamics), inconsistent spatial sampling, and a limited field of view.^34^ These issues are known to be minimized by HD-DOT, but until recently, no wearable HD-DOT technologies had been demonstrated. This is due to the significant engineering challenges associated with building a spatially dense array, with all the associated electronics (sources, detectors, control electronics, power supply, and communications), into a wearable form factor. The first paper to demonstrate a wearable HD-DOT device came with Chitnis et al.,^35^ which showed motor activations in adults using a modular, fiber-less HD-DOT device. Each module contained both source and detector electronics, such that measurement channels are formed both within and across modules. This modular approach allows the creation of dense networks of channels while still allowing the device to conform to the scalp. More recently, Zhao et al.,^36^ using an extension of the same device, demonstrated the feasibility of unconstrained brain imaging in a dual-task paradigm. Following the same modular design principle, von Lühmann et al.^37^ presented a vision of a multimodal wearable design that integrates high-density fNIRS, electroencephalography (EEG), accelerometry, audio recording, and eye-tracking to study brain activity in the everyday world.

Building upon previous research over the last few years,^35^^,^^36^ in this work we applied the first commercially available, wearable HD-DOT instrument (LUMO, Gowerlabs, Ltd.) to image the adult brain. We used a battery of classic visual stimuli protocols to validate and compare the performance of our device against larger fiber-based HD-DOT instruments. The visual cortex has been extensively investigated, and its function is well documented from physiological and electrical studies.^38^ Visual stimulation protocols have been previously used to validate fMRI and PET,^39^^–^^41^ and more recently, to validate fiber-based HD-DOT.^7^^,^^9^^,^^42^ In this paper, we employed lateralized eccentric/peripheral and rotating wedge reversing checkerboard stimuli to map the visual cortex over 15 repeated sessions in one individual. In doing so, we sought to demonstrate the quality of functional imaging that can now be obtained outside of the scanner environment, using a wearable technology and with minimal constraints on the participants.

## 2 Materials and Methods

### 2.1 Wearable HD-DOT System

**Fig. 1:**
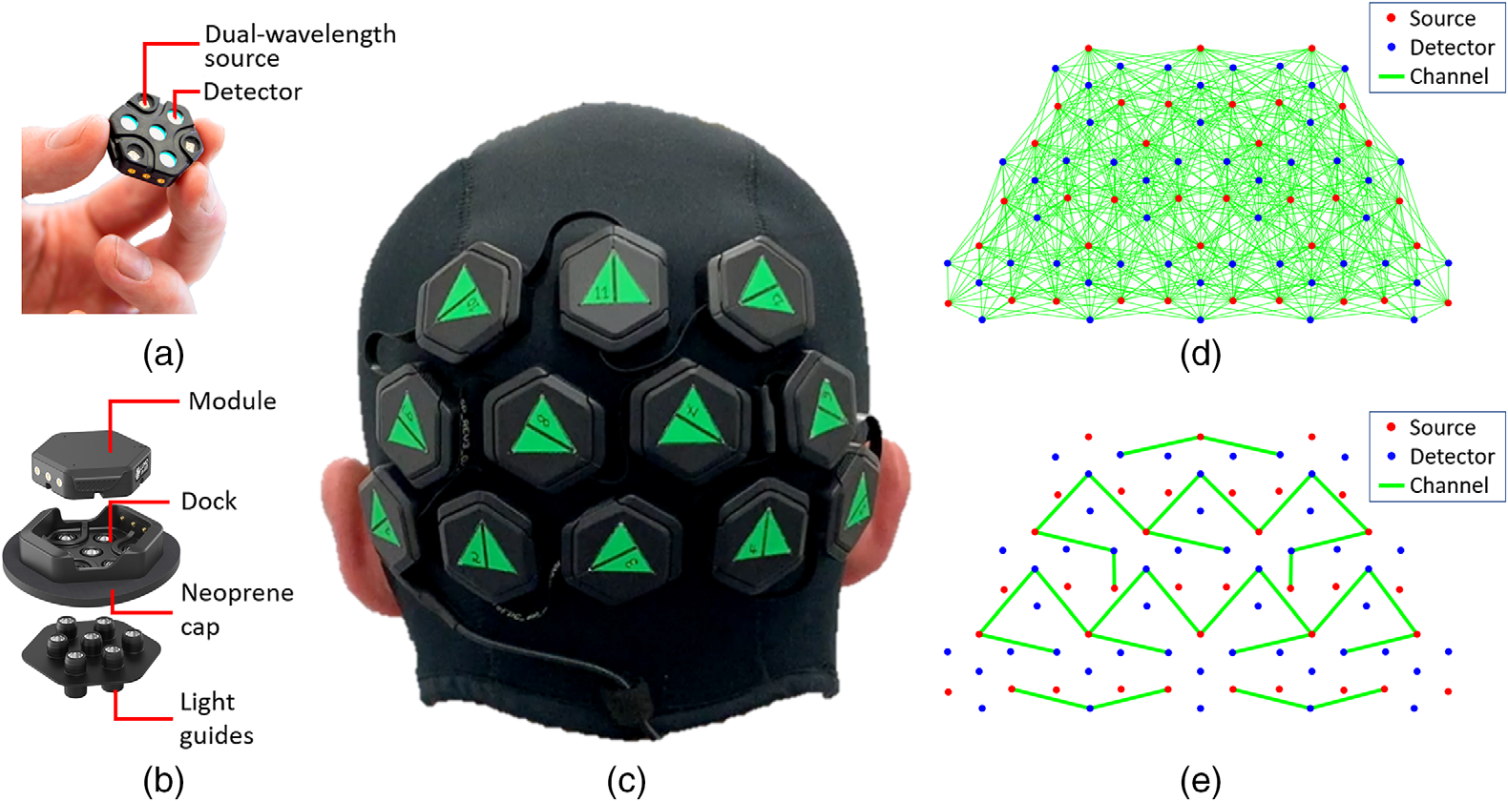
(a) The LUMO tile module; (b) assembly of complete LUMO module showing the tile, dock, and light guides; (c) neoprene cap fitted with a 12-tile LUMO system; (d) high-density array showing channels with source–detector separation ≤40  mm; and (e) sparse array used for comparison against low-density NIRS measurements. The NIRS channels have a source–detector separation of ∼30  mm. Two SS channels 10-mm long on each hemisphere have been included.

The HD-DOT device we applied is a 12-module LUMO system developed by Gowerlabs Ltd., currently intended for research purposes. The system consists of multiple, independent hexagonal modules [or “tiles,” Fig. 1(a)], each containing four photodiodes and three dual-wavelength LEDs emitting at 735 and 850 nm. The tiles are mounted into “docks” [Fig. 1(b)], and a chain of docks is fitted into a neoprene cap. Seven short plastic optical fibers, mounted together to form a “light-guide” carry light from the tile, through the dock and hair to the scalp and back again. This scheme provides extensive flexibility to position the tiles to suit the experimental paradigm while adapting to the curvature of the head. It also makes moving tiles from one cap to another fast and very simple. Figure 1(c) shows a 12-tile array fitted within a neoprene cap, designed to interrogate the visual cortex. The full arrangement yields a total of 1728 dual-wavelength source–detector channels [Fig. 1(d)], which were sampled at a rate of 5 Hz. Of this total number, ∼500 are expected to fall within the 10-to 45-mm range and thus potentially provide viable signals.

### 2.2 Subject and Experimental Protocol

The study considered a single, healthy participant (author R. J. C., male, 36-year old) with normal vision. Fifteen experimental sessions took place over a period of 3 weeks and under lockdown conditions due to the COVID-19 outbreak. The protocol employed was approved by the UCL research ethics committee under application 1133/001; however, in this case, it was performed by the investigator on himself in a home environment. Prior to commencing this study, it was confirmed that this application was outside the remit of the UCL research ethics committee. Each experiment was carried out in a quiet and dimly lit room. First, the participant fitted himself with the neoprene cap and adjusted it with a Velcro chin strap. The cap was positioned to ensure that the same point on the cap (marked with a notch in the neoprene surface) was positioned over the inion, the distance of the cap above the ears was approximately equal on both sides, and the cap’s frontline sat just above the eyebrows. The participant sat in an adjustable chair in front of a 27-in. computer monitor at a viewing distance of 90 cm; the full screen subtended a radial angle of ±12  deg. The participant then started a computer program that controlled the timing of the visual stimuli, provided automated start/stop and synchronization of the LUMO system, and controlled and synchronized a webcam video recording of a front view of the participant.

The video recording of the participant’s face and head was made for quality control and attention monitoring purposes using an inexpensive consumer-grade webcam (1080p, Fusion5 Ltd.). Another researcher (EEVR), not present in the experiment due to COVID restrictions, watched the recording of the full length of the experiment and verified that the participant complied with the protocol, which included: sitting in a relaxed position, maintaining attention to the screen, and remaining awake and alert. An ASCII character was automatically embedded into the video of the participant’s face for synchronization with each stimulus onset, thereby indicating which experimental condition was being shown on the screen at each moment. This helped to verify that the subject maintained attention during the presentation of any given stimulus. No trials were discarded for any session.

**Fig. 2:**
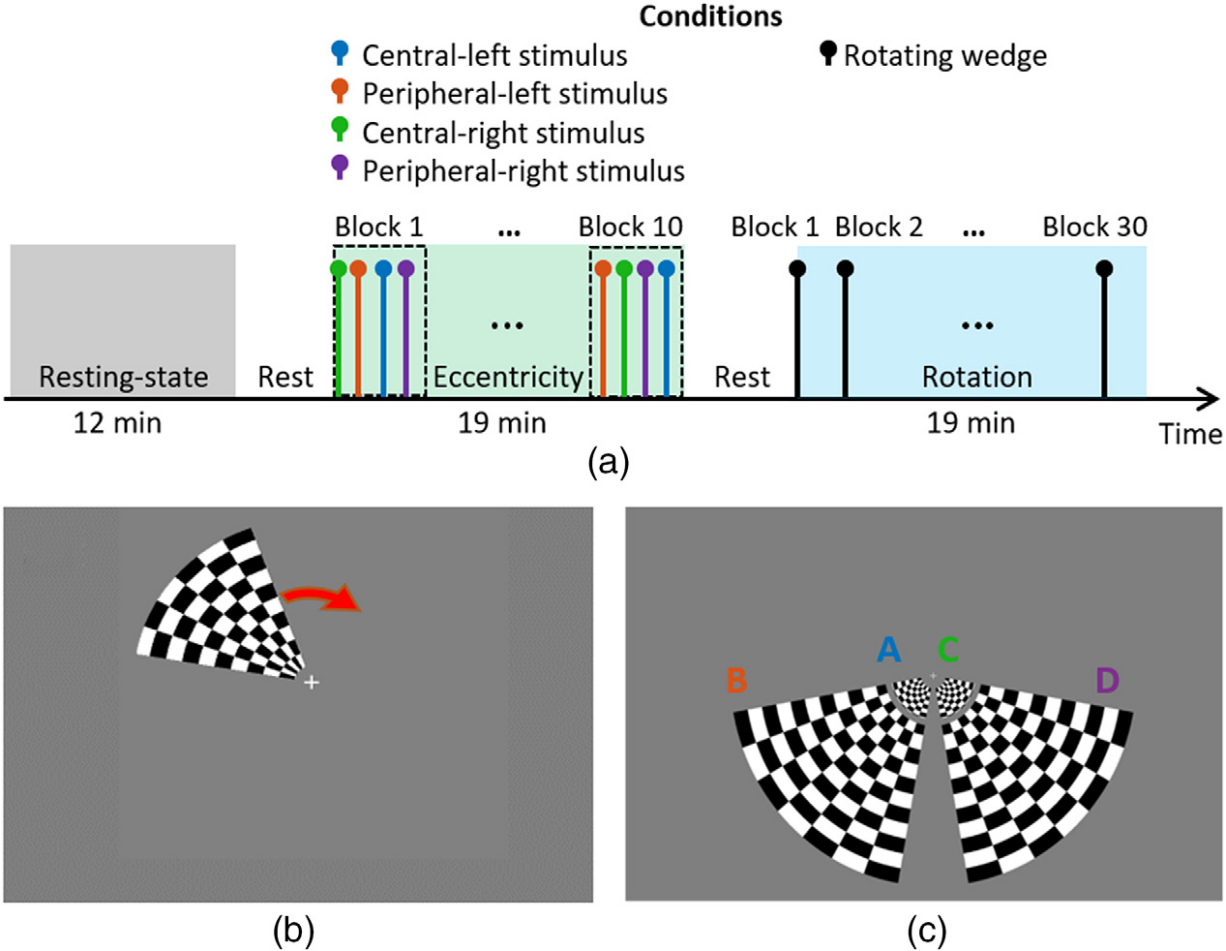
(a) Timeline of the protocol, the rest period is user-defined and lasted <1  min on average. (b) The rotating wedge of the polar angle paradigm used to map the visual field. (c) The eccentricity stimuli, showing the four different radial grids (A, left central; B, left peripheral; C, right central; and D, right peripheral).

The protocol included three paradigms separated by rest periods set by the participant. During the rest periods, the participant was free to move, but did not remove the imaging cap. The first paradigm was 12 min of rest, in which the participant attended to a fixation cross displayed on a 50% gray background. This was followed by an eccentricity mapping and then a polar angle mapping paradigm, each lasting 19 min. The timeline of the paradigm is displayed in Fig. 2(a).

#### 2.2.1 Polar angle mapping

The polar angle mapping paradigm consisted of a radial black and white grid rotating clockwise at 10  deg/s for a full cycle of 36 s [Fig. 2(b)]. The grid extended over a polar angle of 60 deg and a radial angle of 0.5 deg to 10 deg. A baseline period of 45 s was followed by 30 continuous rotations and a return to baseline period of 30 s. This paradigm lasted a total of 19 min.

#### 2.2.2 Eccentricity mapping

The visual stimuli for eccentricity mapping consisted of a black and white radial grid reversing at 10 Hz on a 50% gray background [Fig. 2(c)]. Central and peripheral stimuli were presented, extending over radial angles of 0.5 deg to 1.7 deg and 2.5 deg to 10.5 deg, respectively, and a polar angle of 70 deg for both. An initial baseline period of 15 s was followed by 10 s activation and a resting period lasting a random duration between 15 to 20 s. Each stimulus was repeated ten times in a random order for a total of 40 trials. This paradigm lasted a total of 19 min.

#### 2.2.3 Optode registration

**Fig. 3:**
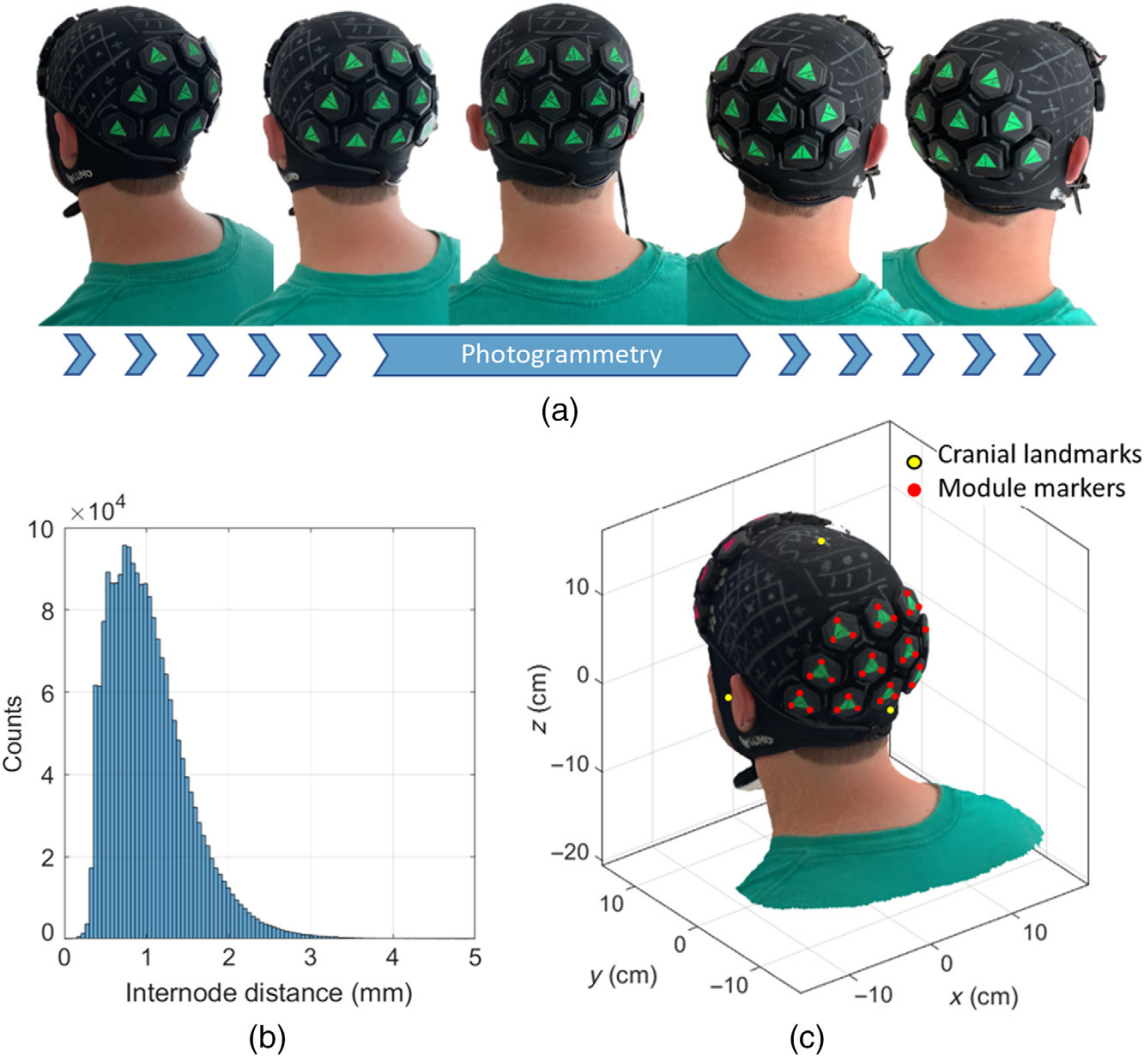
(a) A subset of frames used during the photogrammetry process, derived from a video recording that comprised three full rotations of the head at different heights. (b) Internode distance of the resulting 3D model of the subject’s head. On average, the resolution is <1  mm (c) final 3D surface mesh model of the participant’s head showing the location of head landmarks (yellow circles) and module markers (red circles).

The 3D digitization of the optode locations was performed using photogrammetry. Each LUMO tile has indentations in the upper surface that indicate the position exactly above each of the three sources in the tile. A green fluorescent triangular marker was attached to each tile, with each corner aligned over these indentations. The registration process began by having a second individual record a video of the participant using a smartphone (iPhone XR, Apple Inc.). This video consisted of three panning rotations around the subject’s head at different heights [Fig. 3(a)], whereas the participant was seated and still, with their eyes closed. Each video was ∼90  s in length. The video was directly imported into a commercial software package (Metashape, Agisoft LLC), and between 140 and 200 frames were extracted from the video and used to produce a three-dimensional mesh model in the .ply format that comprised $∼3.5\times 10^{5}$ nodes and $7\times 10^{5}$ faces with a resolution (node spacing) of <1  mm [Fig. 3(b)]. A custom-made program written in MATLAB (MathWorks, Inc.) allowed the manual selection of the locations of the cranial landmarks and tile markers (the corner of each of the green triangles) that were then used to determine the location of the sources and detectors on the subject’s scalp. An example of the participant’s digitized head model is displayed in Fig. 3(c), along with the cranial landmarks (yellow circles) and tile marker positions (red circles). Since the dimensions of the tiles and light guides are fixed and known, the location of the seven optical contact points on the scalp could be computed from the locations of the tile marker positions without further approximation.

### 2.3 Head Modeling and Registration

**Fig. 4:**
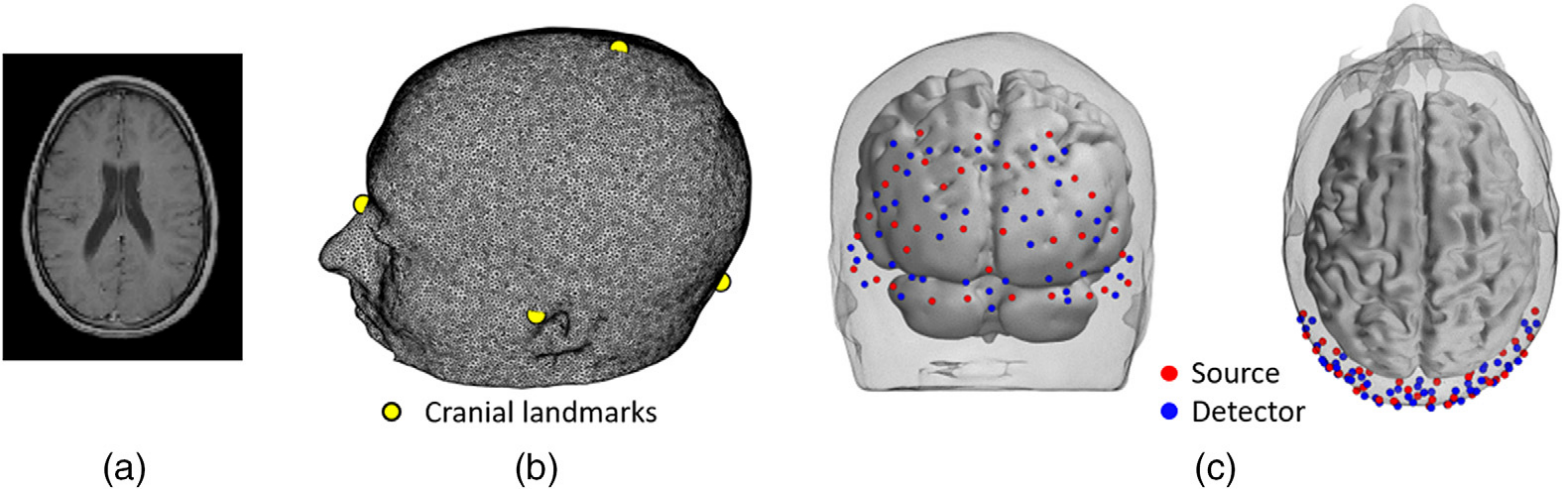
(a) Anatomical MRI; (b) high-resolution tetrahedral mesh, landmarks are indicated with the yellow circles; and (c) 3D model used for image reconstruction. Red and blue circles denote source and detector locations.

**Fig. 5:**
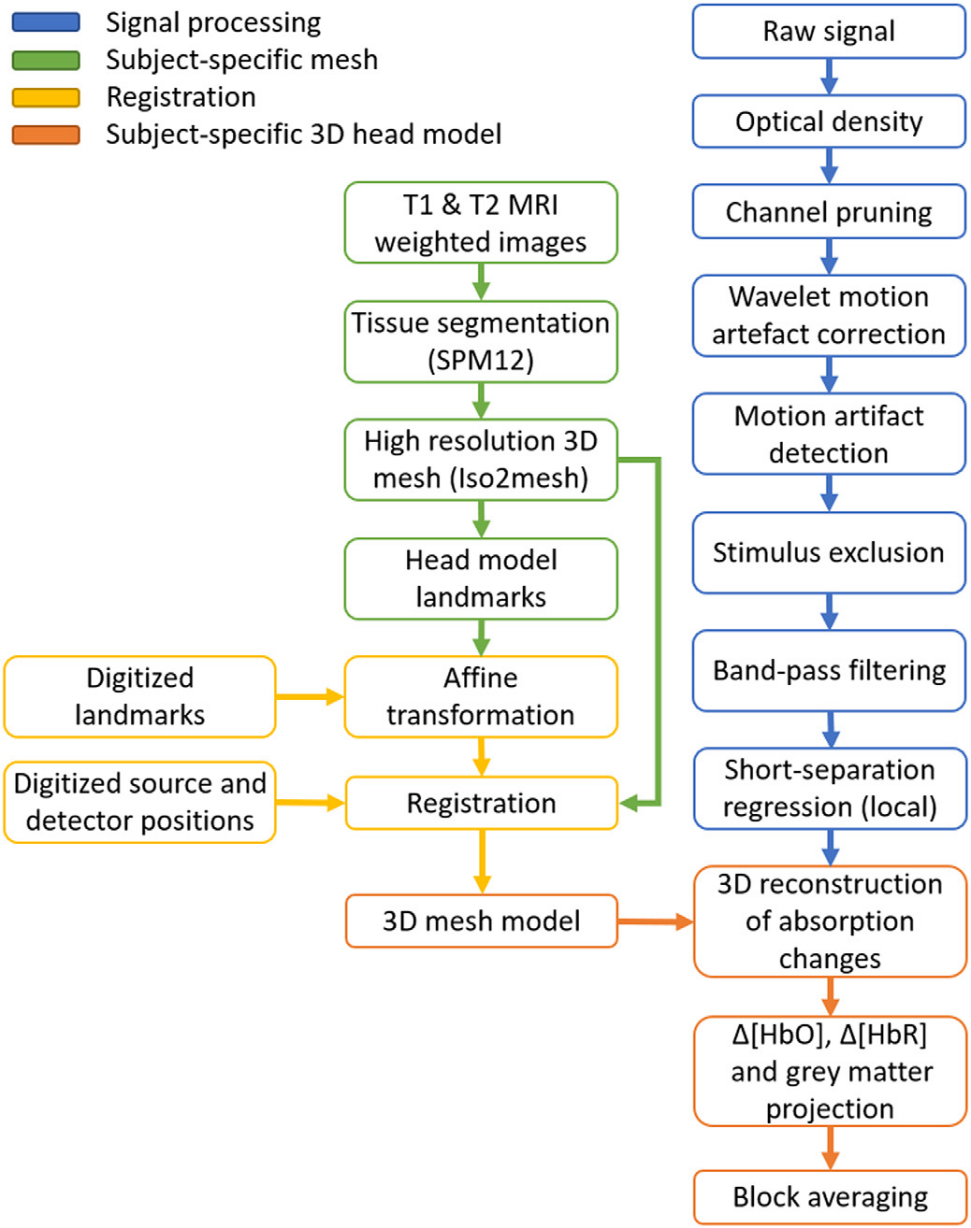
Processing pipeline for head modeling, registration, data preprocessing, and the reconstruction of images of hemodynamic changes.

MRI images of the participant were available from a previous experiment.^36^ The scan consisted of T1- and T2-weighted anatomical MRI images that were linearly co-registered^43^ and used to obtain a five-layer tissue head model using the unified segmentation algorithm.^44^ The head model included scalp, skull, cerebrospinal fluid, gray matter (GM), and white matter tissue layers, which were converted into a high-resolution tetrahedral mesh using Iso2mesh.^45^ The head volume mesh contained $∼2\times 10^{6}$ elements and $∼3\times 10^{5}$ nodes [Fig. 4(b)]. The mesh included the MRI-derived cranial landmarks, which were used to register the digitized source and detector positions into the mesh by means of a rigid transformation. The final 3D model used in the reconstruction stage is shown in Fig. 4(c). Additionally, a surface mesh of the GM was built to aid the visualization of the imaging results. The pipeline for the registration process is illustrated in Fig. 5.

### 2.4 Signal Processing

The data preprocessing pipeline is displayed in blue in Fig. 5. Each step in the pipeline was undertaken using functions from the Homer2 fNIRS processing package^46^ or modified versions thereof. In an initial assessment, channels were discarded based on their coefficient of variation (rejected if mean of intensity/standard deviation of intensity ≤12.5, equivalent to a coefficient of variation >8%). Intensity data were then converted to absorbance (OD), and the remaining channels were motion-corrected using wavelet decomposition with the function hmrMotionCorrectWavelet, with the iqr parameter set to 1.5. Data were then analyzed to detect any residual motion artifacts using hmrMotionArtifact after the correction step. Any remaining artifacts were flagged if the standard deviation changed by a factor >20 within a period of 2 s. If any artifact coincided with the period from 5 s prior to a stimulus onset up to the length of that stimulation block, that stimulus was excluded from further processing. Data from the rotation stimuli were resampled to 1 Hz to obtain a time point per position of the rotating wedge, whereas the eccentricity data were analyzed at the full sampling rate of 5 Hz. Each channel was band-pass filtered using a fifth-order Butterworth filter with low- and high-pass band of 0.025 and 0.5 Hz, respectively.

SS optodes (<15  mm) are mainly sensitive to superficial layers in the adult head, and they have been used routinely to remove the systemic interference present in longer separation channels.^47^ To account for extracerebral contamination, we employed a novel local SS regression approach. Previous work has used a global regression approach, which seeks to remove the signal created by averaging all the SS channels in an array,^42^ or a single SS regression approach that selects an SS channel that is physically closest to the mid-point of the channel in question. Our local SS regression approach consists of regressing the average of the signals derived from all the short channels that share the source or the detector of the channel in question. The rationale behind this approach is that extracerebral contamination of a long-channel signal is primarily due to changes in hemoglobin concentrations directly beneath the source and detector,^10^ and thus the single short channel closest to the mid-point of that long channel is unlikely to be the optimal regressor. To complete the process, the averaged signal of the local SS channels is regressed out from long distance measurements via least squares.

### 2.5 HD-DOT Image Reconstruction

The forward problem was modeled using the perturbation approach where the relationship between measured absorbance changes and optical properties in the medium is expressed as a linear mapping of the form:

$$\Delta A_{\lambda_{i}}=J_{\lambda_{i}}\Delta \mu_{a,\lambda_{i}},$$ (1)

where $\Delta A_{\lambda_{i}}$ is the change in absorbance between the active state and baseline.^48^^,^^49^ For the eccentricity mapping test, the baseline is the rest period immediately prior to the presentation of the stimuli; while for the polar angle mapping experiment, the baseline is the temporal mean of the full time series. $J_{\lambda_{i}}$ is the Jacobian or sensitivity matrix where $\lambda_{i}$ denotes the wavelength (i=1,2), and $\Delta \mu_{a}$ is the change in the absorption coefficient.

The Jacobian matrix was calculated using Toast++, which models the transport of light in tissue using the diffusion approximation.^50^ The numerical solution of the forward problem is obtained using the finite-element method. The optical properties at the wavelengths of interest were linearly interpolated for each tissue using a database of values obtained from the literature.^51^^–^^53^ For efficiency purposes, the Jacobian was calculated in a fine regular grid with size 100×100×100  voxels and projected into the tetrahedral head model.^50^^,^^54^ The changes in absorption coefficient $\Delta \mu_{a,\lambda_{i}}$ were calculated in the voxel space by minimizing an objective function given by

$$\underset{\Delta \mu_{a,\lambda_{i}}}{\arg\;\min}{\left\|\Delta A_{\lambda_{i}}-J_{\lambda_{i}}\Delta \mu_{a,\lambda_{i}}\right\|}^{2}+\lambda F(\mu_{a,\lambda_{i}}).$$ (2)

Due to the ill-conditioned and ill-posed nature of Eq. (2), the problem was solved using zeroth-order Tikhonov regularization. In this case, the functional $F(\mu_{a,\lambda_{i}})$ is reduced to the identity matrix while the regularization hyperparameter λ was set to 0.1 for all the trials. The recovered images of the change in absorption coefficient at the two wavelengths were then converted to images of change in oxy- (HbO) and deoxy-hemoglobin concentrations (HbR) by means of a spectroscopy analysis^55^ and projected to the tetrahedral head model.

### 2.6 Phase Encoded Data Analysis

The polar angle mapping data can be well represented by examining the phase of the oscillating hemodynamic response at the rotational frequency of the rotating wedge stimulus. Phase maps were, therefore, obtained by determining the phase of the oxyhemoglobin signal at the rotational stimulation frequency for each GM mesh node via a Fourier transform of the associated time series. A neurovascular lag of 6.0 s (∼1  radian) between the stimulus and the hemodynamic response was found based on the phase data obtained from the Fourier analysis, which matches the phase lag found in similar HD-DOT experiments^42^ and that reported in other physiological experiments.^56^ Phase maps were therefore corrected by adding this lag to the calculated phase to match the phase maps to the corresponding position of the rotating wedge stimulus. The maps presented followed the convention to define the zero phase as the lower meridian.^42^

### 2.7 Multi-Session Data Analysis

To investigate the repeatability of the hemodynamic response across sessions, two correlation measures were computed. The first is simply the temporal cross correlation of the hemodynamic response functions (HRFs) found at the mesh location of maximum ΔHbO response across all possible pairs of sessions. The second measure provided a spatiotemporal correlation and consisted of concatenating all the HRFs for all the nodes in the field of view (FOV) into a single vector, and then correlating that signal across all sessions in a pair-wise fashion.

### 2.8 Sparse-Array Reconstructions

To directly compare the performance of this HD-DOT device to that of a more typical fNIRS arrangement, we simulated a sparse fNIRS array by selecting a subset of the available channels with source–detector separations equal to ∼30  mm, the standard separation used in adult fNIRS recordings.^5^ As SS channel regression has been shown to reduce the influence of the systemic signal from the extracerebral tissue in fNIRS studies,^10^^,^^47^ it is becoming increasingly commonplace; we also included a pair of channels with source–detector separation of 10 mm; one per hemisphere. The sparse array we considered is shown in Fig. 1(d) and consists of a total of 29 fNIRS channels. Although the geometry of the measurement array prevents us from selecting a regular 30 mm grid array, the simulated sparse array contains almost exactly the same number of channels as a regular 30 mm grid would when applied to the same area. The processing pipeline used to produce sparse-array images followed the exact same steps as in the high-density array, with the exception that SS regression was performed by calculating the mean of the two SS channels, rather than the local regression approach described above.

## 3 Results

### 3.1 System Performance

**Fig. 6:**
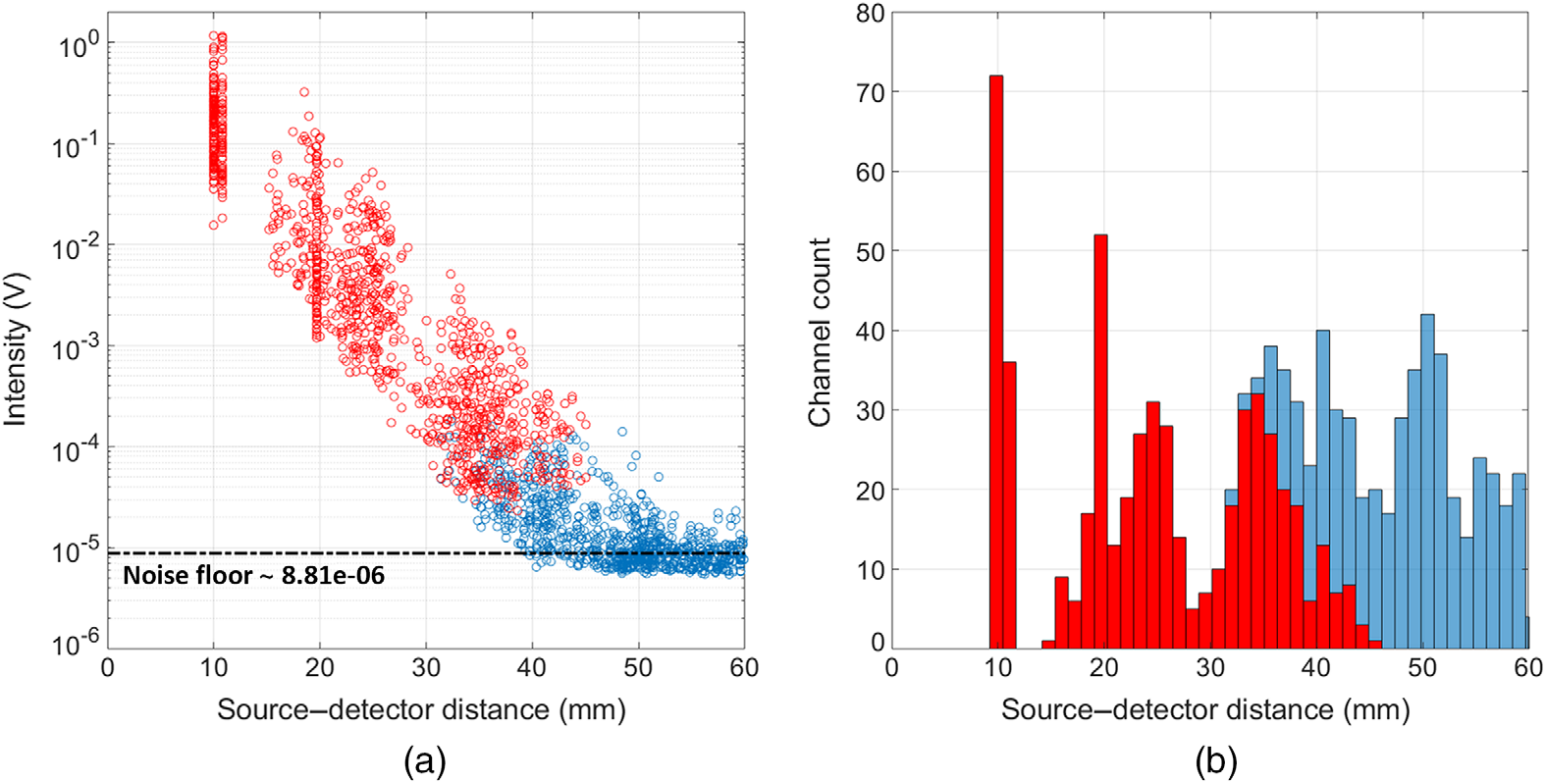
(a) The $\log_{10}$ of the temporal mean intensity values displayed as a function of the source–detector separation at both wavelengths 735 and 850 nm for an example session. (b) The number of dual-wavelength channels available as a function of channel separation.

Figure 6 displays a typical intensity versus source–detector separation scatter plot for this array and participant during a single-rotation stimuli session. Note that data from both wavelengths are included in this plot. Using an estimate of the system noise floor based on the mean signal obtained for channels with a separation >70  mm, the system dynamic range is estimated to be 109 dB, as measured in an *in vivo* experimental circumstance. Though many were above the noise floor of the system, intensity measurements with a high coefficient of variation (>8%, blue circles) were deemed as low quality based on literature norms and were discarded from further processing. For a channel to be passed, both wavelengths had to pass this threshold. For the rotation study, data were resampled to 1 Hz, and on average we kept 100% (114) of the SS (<15  mm) channels, 100% (191) of channels in the range 15 to 25 mm, 80% (136±14) of channels in the range 25 to 35 mm, and ∼34% (47±13 channels) of the channels between 35 and 40 mm. The system therefore provided an average of 489 dual-wavelength channels that were used for further analysis. Note that the equivalent values at 5 Hz (i.e., for the eccentricity data) were marginally worse, with 100% (114), 99% (189±19), 54% (91±13), and 13% (18±9) for each source–detector separation range, respectively, yielding an average of 412 dual-wavelength channels for reconstruction.

### 3.2 Rotation Study

**Fig. 7:**
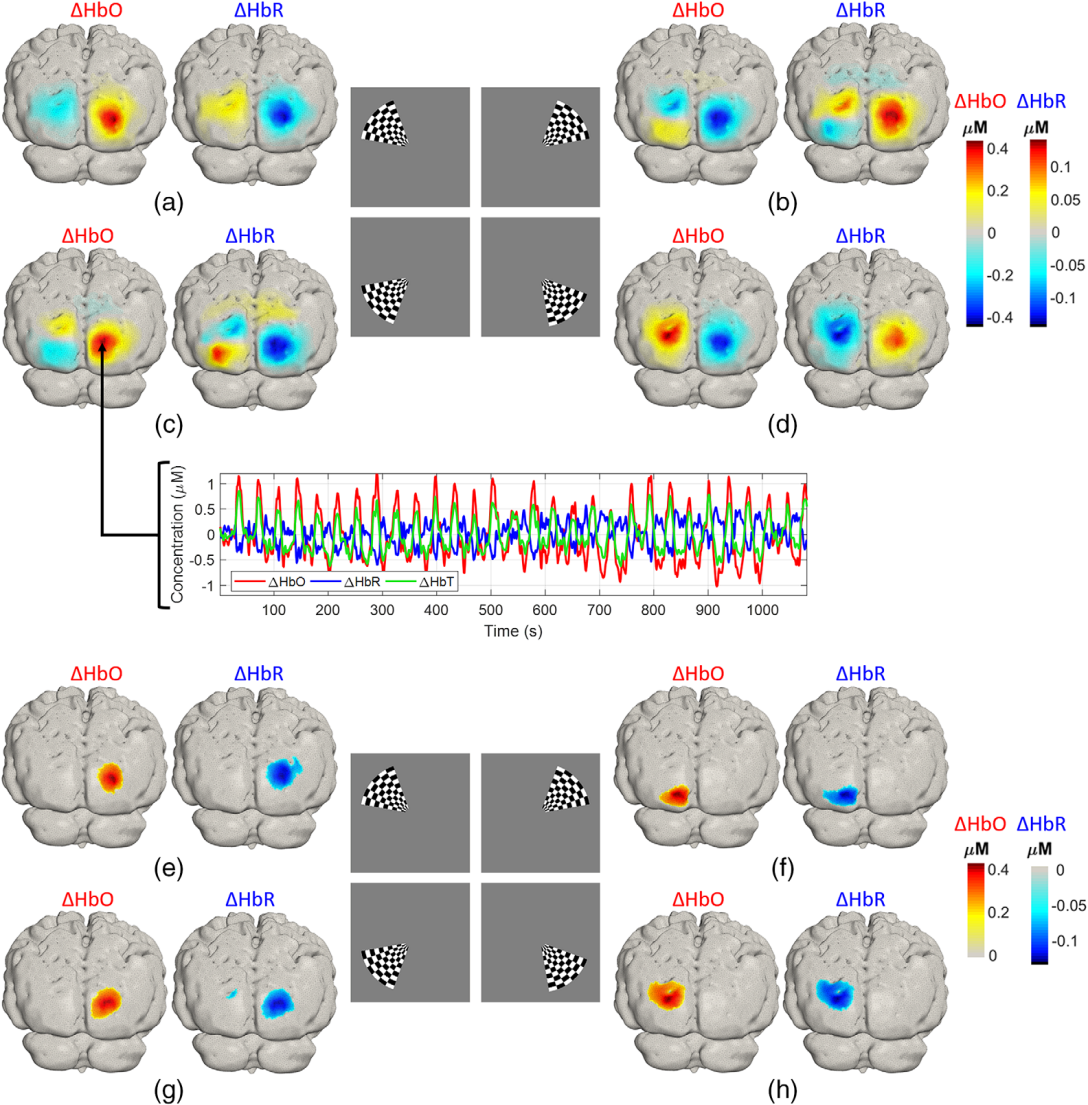
(a)–(d) Example of the activation maps for a single session obtained from the clockwise rotating wedge at each quadrant. Inset in (c) is the time course of the hemodynamic response relative to the average of the full length of the rotation experiment in one single session for the point of maximum ΔHbO in the third quadrant. (e)–(h) Group average ΔHbO and ΔHbR across all 15 sessions, thresholded at 50% of the maximum response.

Three-dimensional reconstructions of oxyhemoglobin changes due to the rotating wedge stimulus for a single recording session are presented in Figs. 7(a)–7(d). The responses corresponding to one frame per quadrant are displayed (at −45  deg, 45 deg, 135 deg, and 225 deg). As expected, the activations are contra-lateral to the stimuli. Note that the maximal change occurs in the upper visual cortex [Figs. 7(c) and 7(d)] corresponding to the stimulation in the lower visual field [Figs. 7(c) and 7(d), inset]. Each positive change in ΔHbO contralateral to the stimulus is accompanied by a corresponding negative contrast in the opposite hemisphere, ipsilateral to the stimulus. However, this does not imply a negative response. Rather, it is simply the result of reconstructing images relative to the average of the data across each whole block (30 rotations), areas that show a large activation for a given stimulation angle (ϕ), will show an inversion (a “shadow”) of that response for (ϕ+π). The negative change in ΔHbO found in each case is the approximate inverse of the positive response found for the opposite (antiphase) stimulus. The same pattern is evident in ΔHbR images, particularly for panel (b)—the upper right quadrant visual stimulation. This stimulus yielded the smallest contralateral functional response and the images are therefore dominated by “shadows” from the lower left quadrant stimulation (panel c). The time series in the inset displayed in Fig. 7(c) are the time-averaged responses at the node with maximum ΔHbO in a single session, note the strong contrast each time the rotating wedge passed this point. Figures 7(e)–7(h) show similarly organized figures but show the average across all 15 sessions, thresholded at 50% of the peak change.

**Fig. 8:**
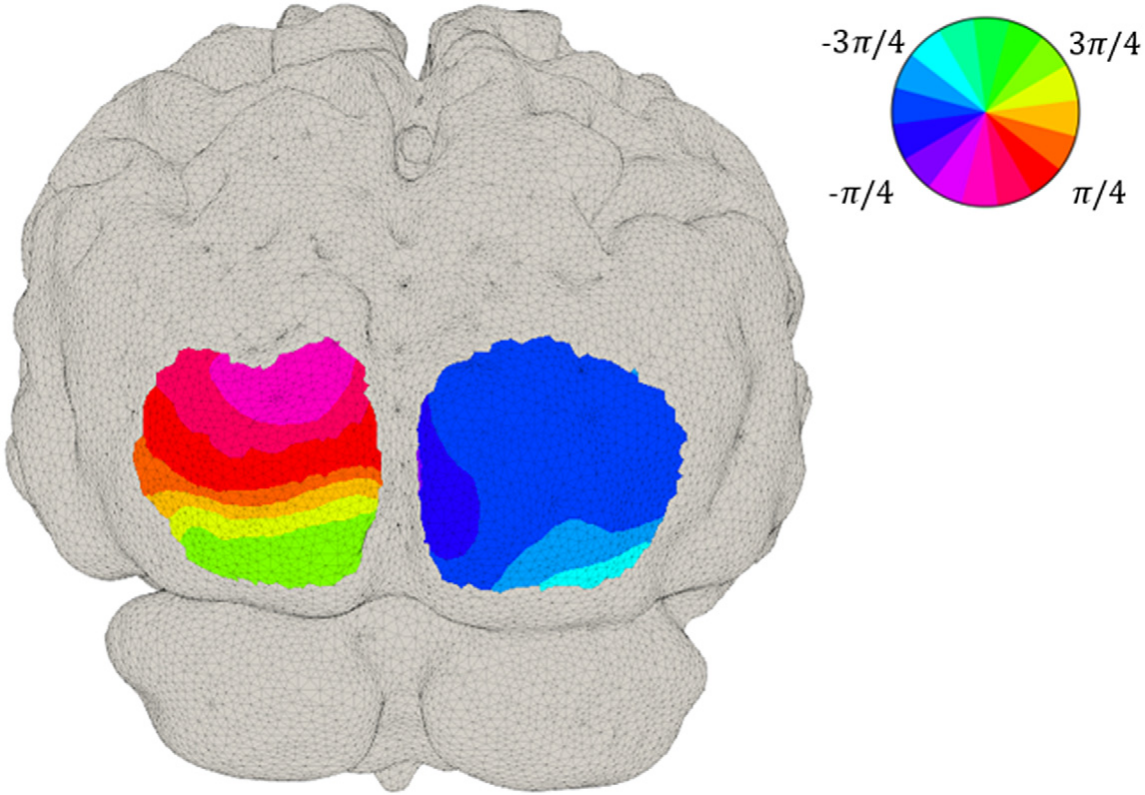
Phase data obtained from the Fourier transform of the time series at each node location at group level. The phase corresponds to the delay between the start of the stimulus and the activation in each region of the cortex.

The phase map presented in Fig. 8 is a depiction of the relationship between the visual angle (the position of the wedge) and the corresponding area in the visual cortex. Phase values were obtained from the Fourier transform of the time series at each node location, following a correction of ∼1  radian to account for the neurovascular lag.^42^ Note that the phase maps demonstrate clearer phase contrast in the upper visual cortex, where responses had superior SNR.

### 3.3 Eccentricity Study

**Fig. 9:**
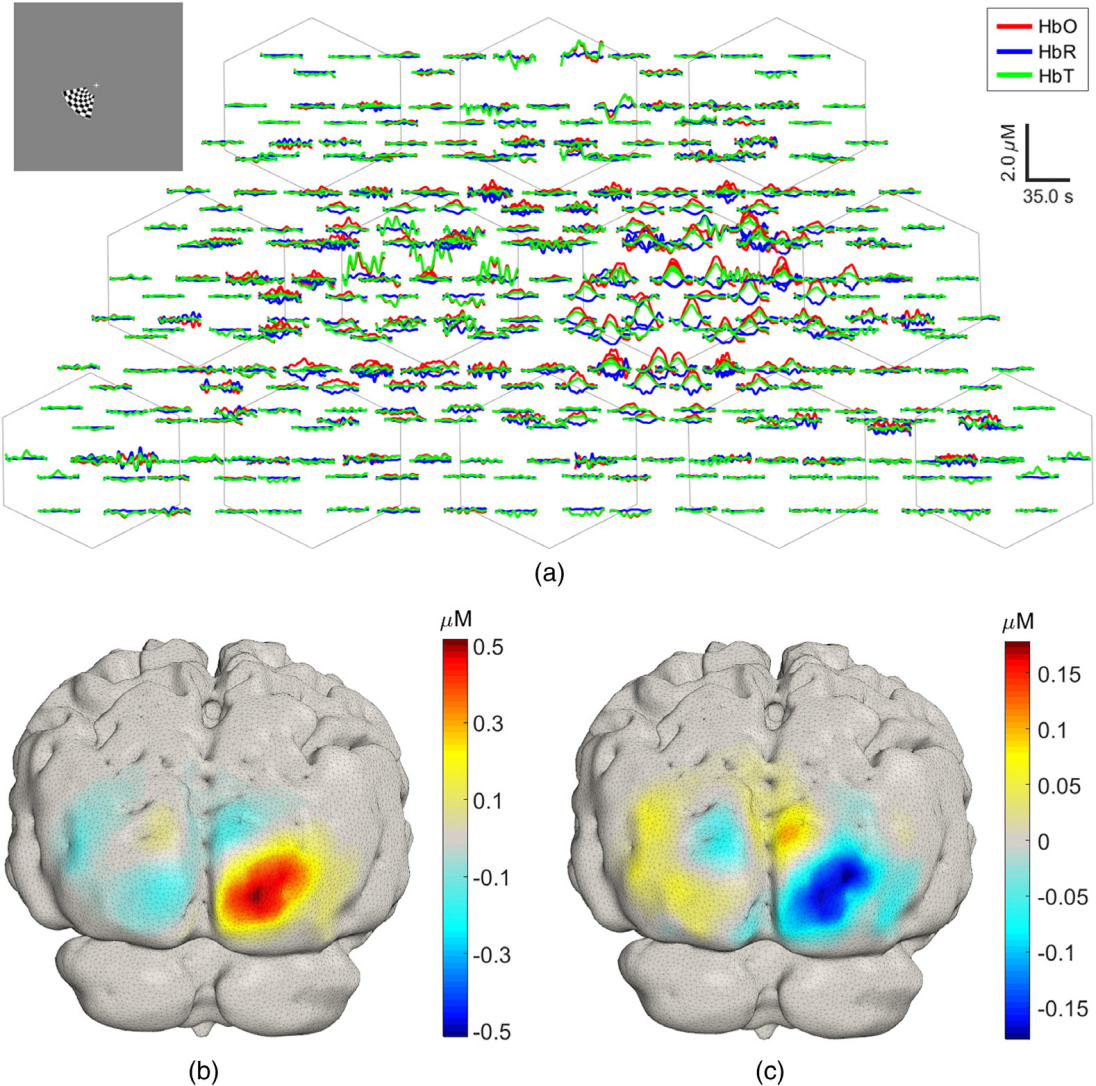
(a) Example single-session HRFs for a left central stimulus calculated using the block-average approach and plotted in the channel space. The stimulus is displayed in the inset on the top left corner. (b), (c) The corresponding reconstructed ΔHbO and ΔHbR image projected into the GM mesh.

An example hemodynamic response shown in the traditional representation of fNIRS data (“channel space”), due to visual stimulation of the lower left central region of the visual field, derived from a single session, is displayed in Fig. 9(a). The central-left stimulus, shown inset, produces an increase of oxyhemoglobin on the contralateral channels, with some smaller amplitude activations also present in the ipsilateral hemisphere. Figures 9(b) and 9(c) show the 3D reconstructions of ΔHbO and ΔHbR, displayed on the subject-specific GM mesh. Large hemoglobin concentration changes are located in the contralateral (right) hemisphere, with some residual increases and relative decreases in ΔHbO in the left hemisphere.

**Fig. 10:**
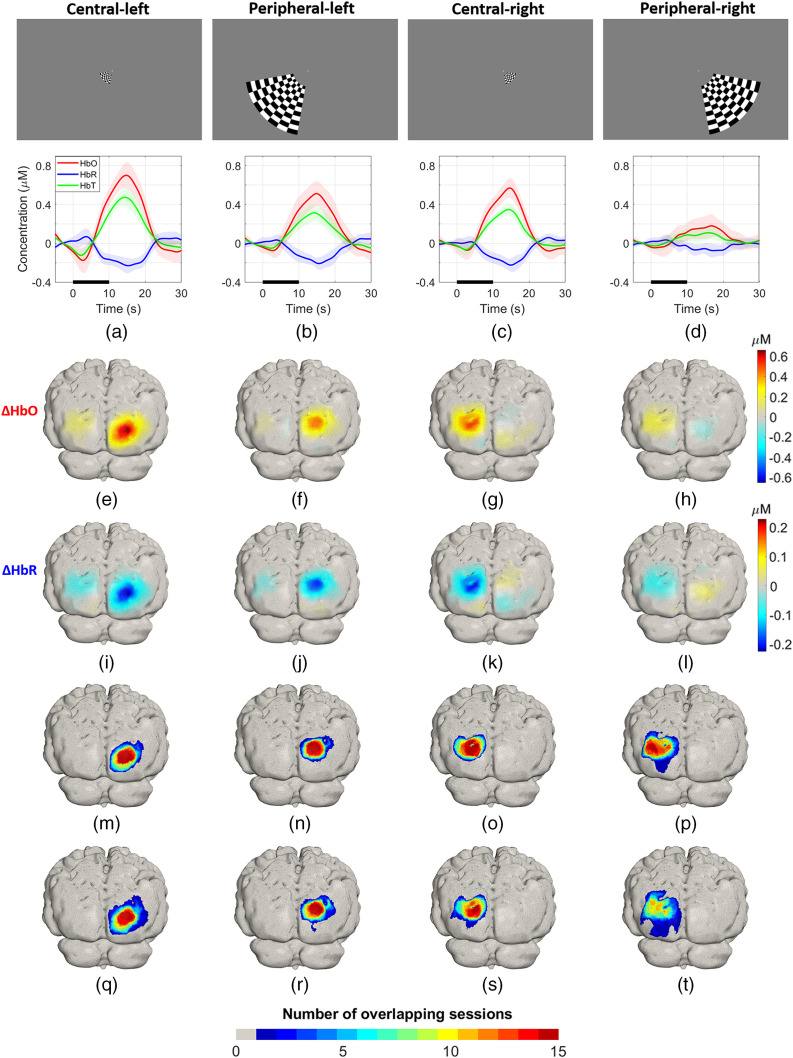
(a)–(d) Upper panels: stimuli used for eccentricity experiment. Lower panels: group average HRF for each stimulus. The thick line indicates the mean HRF at the node with the highest response and the shaded area indicates the standard deviation on that mean across sessions for each of the four conditions. (e)–(h) and (i)–(l) Group average ΔHbO and ΔHbR across all sessions for each condition, respectively. (m)–(p) and (q)–(t) Overlap of activations ΔHbO and ΔHbR for each condition.

Figures 10(a)–10(d) show in the lower panels the group average HRFs, where the activation contrast for all the conditions is robust and clearly distinguishable from the baseline or recovery period. The peripheral-right condition showed the smallest concentration changes and the highest variability. Figures 10(e)–10(h) and 10(i)–10(l) are the grand average ΔHbO and ΔHbR images, respectively, for each condition. The central activations achieved higher concentration change values in comparison to the peripheral stimuli. In particular, the peripheral-right activation is ∼70% smaller in amplitude than the maximum (central-left) response. Changes in ΔHbO and ΔHbR are co-located but of opposite polarity, as is expected. On average, the absolute magnitude of ΔHbR is ∼40% of ΔHbO for all the conditions $({\left|\Delta \mathrm{HbR}\right|}_{\max}/{\left|\Delta \mathrm{HbO}\right|}_{\max}\times 100=A$: 34.3% and B: 42%, C: 38.8%, D: 43.2%).

Figures 10(m)–10(p) show the sum of binary maps of ΔHbO response across sessions for each condition. Each map therefore represents the number of sessions, in which a node location lies within 50% of the maximum ΔHbO response. These distributions show clear areas, in which the response was present in every one of the 15 repeated sessions, illustrating the consistency of these images across sessions. The area where a response was present in at least seven of the sessions occupies a large proportion of the area, for which there was a response in at least one session: for conditions A to D, these proportions were 53%, 48%, 67%, and 49%, respectively, averaging 55%. For the area showing a response in at least 14 out of 15 sessions, the proportions are 19%, 20%, 26% and 7%, with an average of 18%. Figures 10(q)–10(t) are the equivalent overlap maps for ΔHbR for each condition. The regions are of similar extent, and with the exception of the peripheral-right response, show areas that were active in every one of our 15 sessions. However, the area associated with only one or two overlapping sessions is clearly larger than that for ΔHbO. The proportions of the area that shows a response in at least seven sessions relative to the area where at least one session causes a response are 35%, 43%, 50% and 28% for conditions A to D, respectively; and the average is 39%. For 14 out of 15 sessions, the equivalent proportions are 12%, 14%, 11%, and 0%.

**Fig. 11:**
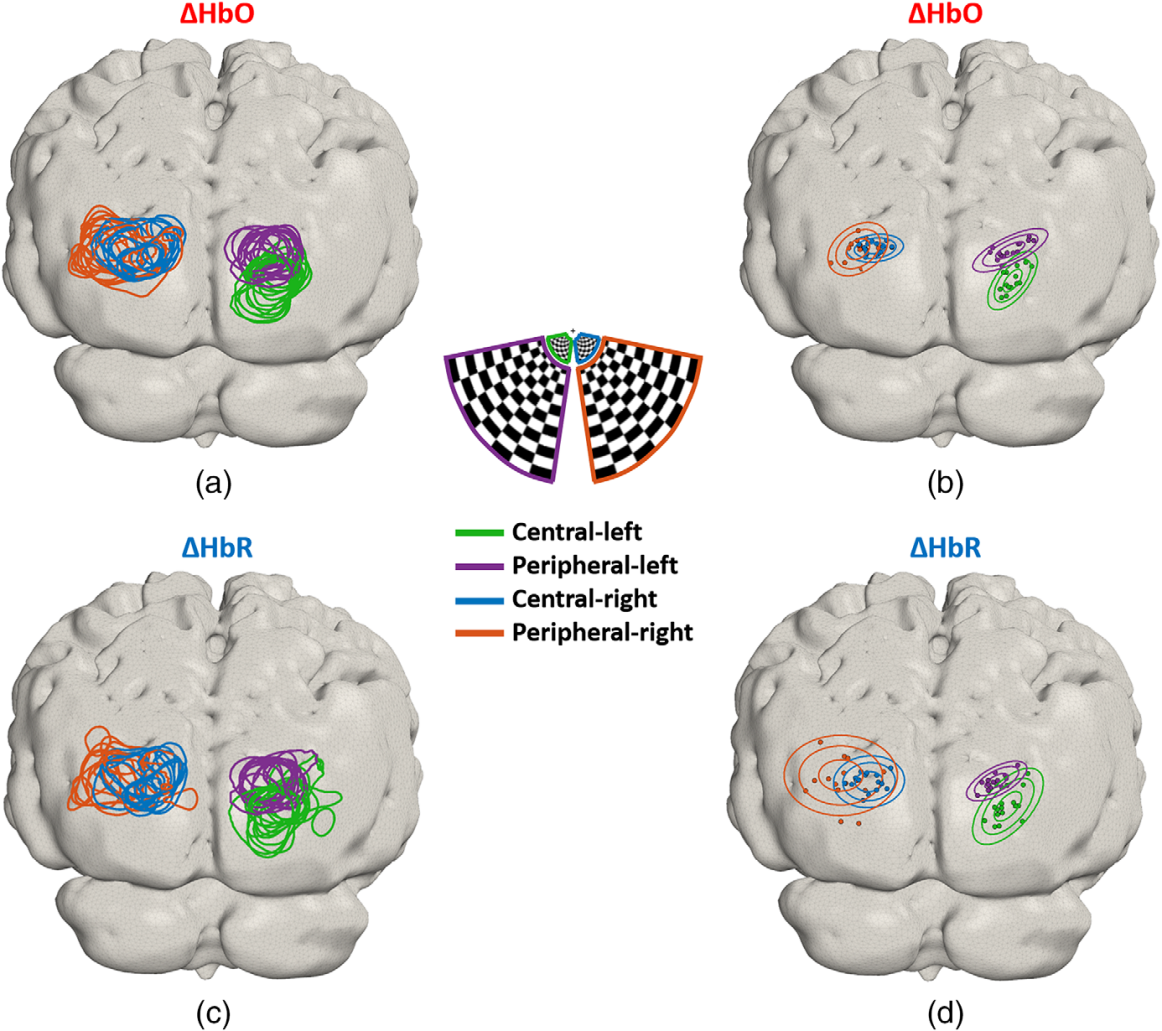
(a) Contours at 75% of maximum ΔHbO for each session and (b) the location of maximum ΔHbO increase per session is indicated with the filled circles. Color denotes condition. The elliptical curves are the contours of the 3D Gaussian probability density functions at one, two, and three standard deviations. Similarly, (c) each line is the contour at 75% of minimum ΔHbR for each session. (d) The location of the minimum ΔHbR per session is indicated with filled circles. Each condition is color coded as indicated in the legend.

To compare the location of the activations across different sessions for all the conditions, Fig. 11(a) shows the contours calculated at 75% of the maximum change in ΔHbO for each condition and for each session. Four distinct regions of response can be distinguished, with a larger overlap between responses on the left hemisphere (peripheral and central-right stimuli). Fig. 11(b) shows the location of the maximum ΔHbO value for each session and the contours of a 3D Gaussian distribution at one, two, and three standard deviations ($\sigma_{x}=2.2$, 3, 3.9, and 5.2, $\sigma_{z}=3$, 2.1, 2.2, and 2.9 for conditions A to D). Note that the peak responses are constrained to a relatively small region in each case. Similarly, Figs. 11(c) and 11(d) show the corresponding images for ΔHbR. Note that the shapes of the contours at 75% of the minimum are less uniform and spread over a larger area, which is also supported by the larger dispersion of the 3D Gaussian distributions shown in Fig. 11(d), likely due to the lower contrast of ΔHbR.

The average horizontal shift between the peak ΔHbO response to the left and right central stimuli across all sessions is $\Delta x_{\mathrm{AC}}=44\pm 5\;\mathrm{mm}$; whereas in the vertical direction, it is $\Delta z_{\mathrm{AC}}=-10\pm 3\;\mathrm{mm}$. For the central-left and peripheral-left, the horizontal shift is $\Delta x_{\mathrm{AB}}=-2\pm 2\;\mathrm{mm}$, and the vertical shift is $\Delta z_{\mathrm{AB}}=9\pm 2\;\mathrm{mm}$. The horizontal shift between central-right and peripheral-right responses is $\Delta x_{\mathrm{CD}}=-9\pm 6\;\mathrm{mm}$, whereas the corresponding shift in the vertical direction is $\Delta z_{\mathrm{CD}}=-1\pm 3\;\mathrm{mm}$.

**Fig. 12:**
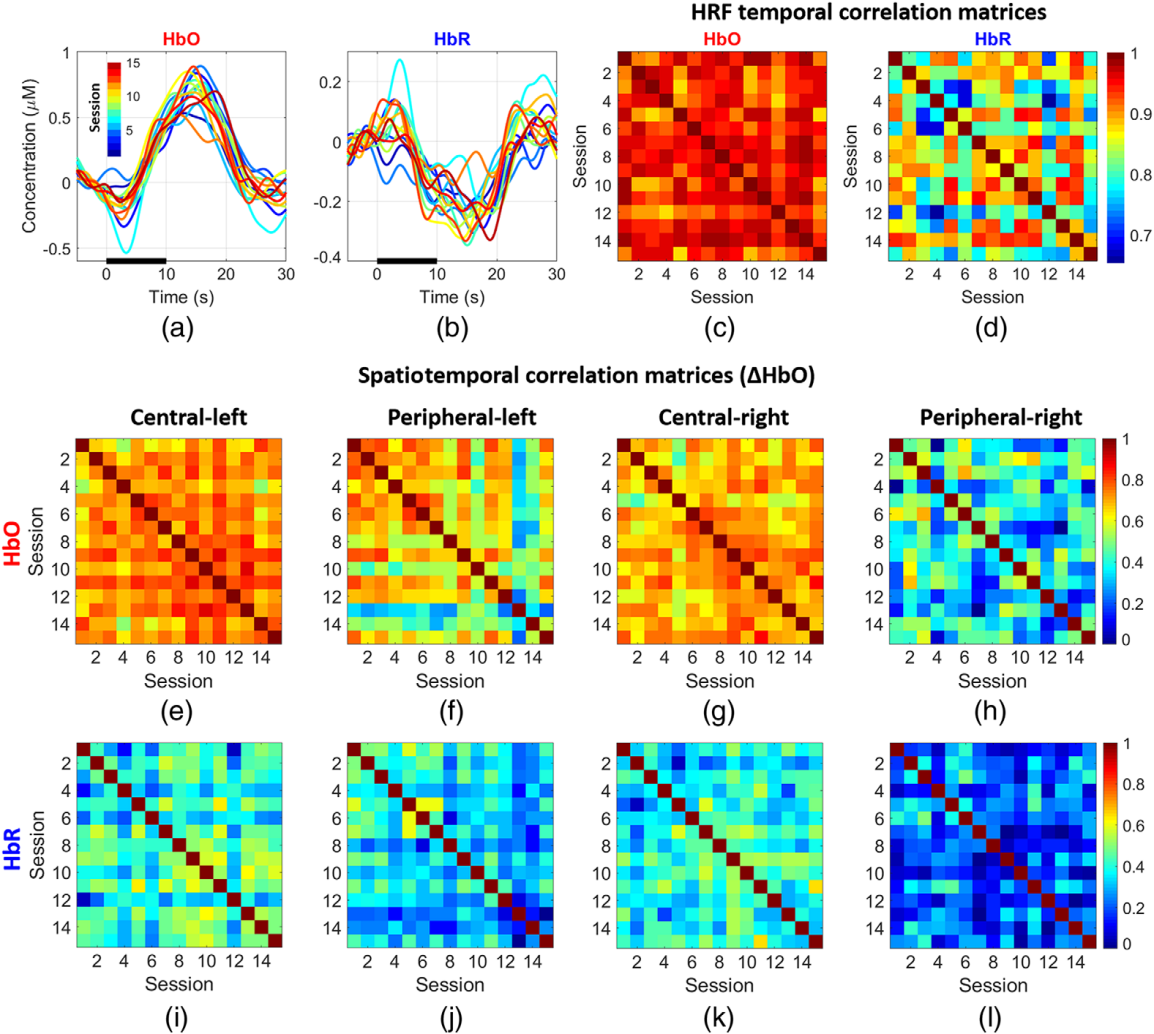
(a), (b) Temporal changes of maximum oxy- and deoxy-hemoglobin for the central-left stimulus. Each curve represents a session. (c), (d) The temporal correlation matrix of the HRF at the peak node for each session for ΔHbO and ΔHbR, respectively. Note that the responses across all sessions are very similar; the average Pearson correlation coefficient is 0.95. (e)–(h) Spatiotemporal correlations for all sessions for ΔHbO: each element in the correlation matrix was calculated by correlating the HRFs for all node locations across all 15 sessions. Similarly, panels (i)–(l) are the spatiotemporal correlations at group level for ΔHbR.

Figures 12(a) and 12(b) depict the HRFs for the central-left condition at the node associated with the maximum change in HbO for ΔHbO (a) and ΔHbR (b), respectively. Note the high degree of similarity among all the curves across sessions for both chromophores. The consistency of the response is further confirmed by the HRF temporal correlation matrices displayed in Figs. 12(c) and 12(d), which show the temporal correlation between the average HbO (c) and HbR (d) response curves for the central-left stimulus at the peak node for each session and that of every other session. All these values are remarkably high. For HbO, r is always >0.87 and for HbR r is always >0.65. Figures 12(e)–12(h) are the spatiotemporal correlation matrices for ΔHbO, where each element is the correlation between the concatenated time series of the responses at all nodes across two given sessions. Activations due to central stimuli achieved higher correlation values than the peripheral stimuli, with the peripheral-right stimuli having the lowest correlations, overall, the correlation value is around R∼0.7 except for the peripheral right condition which is R∼0.5. Similarly, Figs. 12(i)–12(l) show the group spatiotemporal correlations for ΔHbR, note correlation is still evident albeit with lower values (typically around R∼0.4 for conditions other than peripheral right). This is expected given the lower SNR of the ΔHbR changes.

### 3.4 Sparse-Array and High-Density Reconstructions

**Fig. 13:**
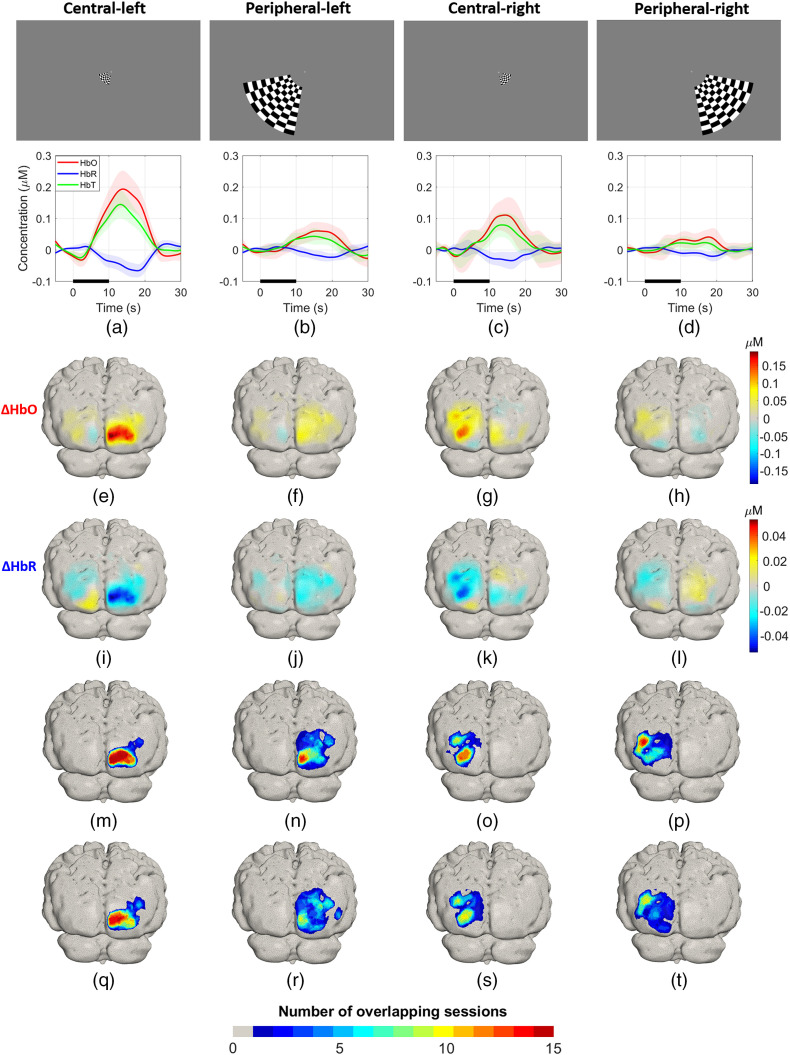
(a)–(d) Upper panels: stimuli used for eccentricity experiment. Lower panels: group average HRF obtained with the sparse array indicated in Fig. 1(e). The thick lines indicate the mean ΔHbO, ΔHbR, and ΔHbT across all repetitions, and the shaded bands indicate the standard deviation. (e)–(h) and (i)–(l) Group average ΔHbO and ΔHbR across all sessions for each condition, respectively. (m)–(p) and (q)–(t) Overlap of activations ΔHbO and ΔHbR for each condition.

Figures 13(a)–13(d) (lower panels) show the group average hemodynamic responses obtained with the simulated sparse array, at the node with the largest HbO response. Note the lower amplitude of the activations in comparison with those obtained for the high-density array displayed in Figs. 10(a)–10(d), especially for the peripheral-right condition. Figures 13(e)–13(h) are the recovered changes in ΔHbO for each condition, respectively. In general, while the peak changes are broadly lateralized to the correct hemisphere, the responses are noticeably more diffuse than those of Fig. 10 (e.g., panel f), the peak change often occurs at a different cortical location (e.g., panels e–g), and in some cases the images demonstrate multiple separate foci (e.g., panels g and j). The magnitude of the increase in ΔHbO is also consistently lower than that of the HD-DOT array, which provides on average fivefold larger amplitude (ratio of max $\Delta {\mathrm{HbO}}_{\mathrm{HD}\text{-}\mathrm{DOT}}$ to max $\Delta {\mathrm{HbO}}_{\mathrm{LD}\text{-}\mathrm{fNIRS}}=3.6$, 8.5, 5.1, and 5.1 for conditions A to D). Also, on average, the HD array doubles the SNR provided by the low-density array (1.8, 1.9, 2.9, and 1.3 for conditions A to D, respectively). Figures 13(i)–13(l) show reconstructed changes in ΔHbR where similar features to the ΔHbO images can be seen. Again, the contrast observed with the low-density array is noticeably lower than that of the HD-DOT array. The HD-DOT ΔHbR images provide an SNR that, on average, is twice as large as the low-density equivalent (1.4, 2.3, 2.7, and 2.5 for conditions A to D, respectively).

Figures 13(m)–13(p) show the overlap maps for ΔHbO. Only the response to the central-left condition yields a region that was active in all 15 sessions, though the centre-of-mass of that response is clearly off-set in comparison to that of Fig. 10(m). The overlap maps for the other conditions show poorer performance. The proportions of the area that shows a response in at least seven sessions relative to the area where at least one session causes a response are 50%, 17%, 30% and 24% for conditions A to D, respectively, yielding an average of only 30%. These percentages are lower for ΔHbR: 43%, 9%, 21%, and 12% for conditions A to D, respectively, averaging 21%.

## 4 Discussion

Fiber-based HD-DOT has demonstrated a resolution and accuracy approaching that of blood oxygen level dependent-fMRI in the adult brain.^9^^,^^13^ In this work, we sought to demonstrate that comparable imaging quality is now achievable in a wearable form factor. We introduced a new HD-DOT system (LUMO, Gowerlabs, Ltd.) that is built upon a modular design architecture and provided dense DOT sampling in a compact and ergonomic form. We demonstrated the performance of the system by replicating a series of classical visual paradigms which have been previously validated with fiber-based HD-DOT^7^^,^^42^ and undertaking test–retest comparisons in a single, extensively imaged individual. Although this N=1 study can say nothing about the performance of this device across a population, our results show that the location accuracy, magnitude, and spatial extent of the activation maps that can be obtained with this device are broadly comparable to those of larger, fiber-based HD-DOT systems, and that such measurements can be obtained with relative ease, even in a home setting.

### 4.1 System Performance

An earlier example of a modular HD-DOT device was presented by our group in Ref. 35, which included four square modules with all the optics and the majority of the acquisition electronics integrated within them. These 35 mm modules were daisy-chained together and connected to a central on-bench control unit. This design yielded the first functional images obtained with a fiberless, HD-DOT system. Earlier in 2020, an extended version of this “micro-NTS” system was used to demonstrate functional imaging capability during a walking paradigm.^36^

The system presented here represents a fundamental redesign of a modular, wearable HD-DOT technology. The aim of this study was to validate the capabilities of this new system. Key to this is the dynamic range—which relates to the range of source–detector separations that are obtainable. Figure 6(a) shows the initial quality assessment applied to our data. Using an estimate of the system noise floor based on the mean signal obtained for channels with a separation >70  mm, and given the known measurement ceiling, the system dynamic range exceeds 100 dB, as measured in an *in vivo* experimental circumstance. The red circles indicate channels with SNR>12.5. Data that were of sufficient quality to be used in image reconstruction were obtained across a broad range of source–detector distances, from 10 to 45 mm. In this paper, we did not precisely characterize the device performance using laboratory-based testing and phantom measurements. Instead, we chose to pursue a practical assessment of performance based on *in vivo* measurements in a non-laboratory setting. The quality of the data shown in Fig. 6 (spanning multiple source–detector distances) demonstrates the utility of this device, and that it can be applied easily in a real experimental scenario.

### 4.2 Polar Angle Mapping

Polar angle mapping is a classic cortical imaging task that was pioneered by Engel et al.^40^^,^^41^ to study the visual cortex non-invasively. In this study, retinotopic mapping of the phase stimulus was made possible due to the large number of measurements available in an FOV that covers a large portion of the visual cortex (Fig. 1). Figures 7(a)–7(d) show the activation maps corresponding to the rotating wedge stimulus being present in each quadrant of the visual field. Note that for each frame, the response is always maximal in the opposite quadrant in relation to the stimulus. The large negative ΔHbO in the opposite quadrant does not imply a decrease in HbO in response to this stimulus, but is effectively an artifact of the reconstructions being relative to the average of the signal over the full block.^7^^,^^9^^,^^42^ The largest activation in Fig. 7 is located contralateral to the stimuli in three out of four quadrants for the single-session results; however, the grand-average results shown in Figs. 7(e)–7(h), correctly locate the largest activation for all the conditions in the contralateral hemisphere to the stimulus. Similar results were obtained in the eccentricity study, as we explain below, where the activations in the right hemisphere showed smaller contrast compared to those obtained in the left hemisphere. The inset in Fig. 7(c) shows the time trace of HbO, HbR, and HbT for this single selected session. Note that clear responses are evident to individual stimuli with remarkably large contrast evident each time the rotating wedge activates this region.^9^

Figure 8 shows the phase map associated with these cortical activations, note the smaller spatial extent of the regions corresponding to the upper visual fields; this is consistent with previous observations.^42^ These results are potentially due to the system achieving poorer sensitivity where the lower visual cortex folds inward, away from the array. Retinotopic phase mapping of this form requires a continuous map of coded phases, each one corresponding to a visual angle, and thus requires spatial continuity and high sensitivity across the visual cortex. To date, visual cortex studies using traditional fNIRS have been mostly limited to differentiating activation versus rest or left or right lateralization.^57^^–^^61^

### 4.3 Eccentricity Mapping

High-density diffuse optical imaging relies on a large channel count to achieve densely spaced overlapping samples and provide increased spatial resolution and accuracy. Figure 9(a) highlights the dense number of measurements in the channel space provided by this system, which contributes to the robust reconstructed activations shown at the single-session level in Fig. 9(b). The maximum observed change in HbO is around 0.5  μM, and there is some more diffuse activation in the hemisphere ipsilateral to the stimulus. This is similar to responses that have been obtained with fiber-based systems.^7^^,^^42^

Our test–retest results provide strong evidence that the HD-DOT system introduced in this study can recover activations over multiple sessions very reliably. This is despite the removal and reapplication of the cap for each session, and the fact that in this case the cap was self-applied. The group average HRFs displayed in Figs. 10(a)–10(d) (lower panels) show that the activations are robust, and the contrast is high in comparison to the baseline and rest periods. Additionally, the small standard deviation apparent on the HRF curves shows that the responses are consistent across all sessions, which is further confirmed by the correlation analyses shown in Fig. 12. The group average ΔHbO and ΔHbR maps displayed in Figs. 10(e)–10(l) show robust concentration changes on the contralateral hemisphere to the visual stimuli in both chromophores.

The overlap maps for ΔHbO and ΔHbR shown in Figs. 10(m)–10(p) and Figs. 10(q)–10(t), respectively, highlight the high degree of consistency and repeatability for all the conditions across sessions; only the peripheral-right condition shows larger spatial and temporal variability. Figure 11(a) shows that the region of the activation for the peripheral-right condition spans a larger area than the other conditions, and the activation is more diffuse and overlaps extensively with the central-right stimulus. Given the symmetry of the array, we have no reason to suspect that the weaker response to the central-right stimulus is due to any technical limitation of the instrument. Rather, we suspect that the hemodynamic response to the peripheral right stimulus was genuinely smaller in this subject. This is supported by the fact that the response to the stimulus was consistently lower in amplitude than the other three conditions across all 15 sessions, despite the inherent variation in the exact positioning of the array from session to session. This hypothesis is also supported by the rotating wedge experiment, where the phase map is not fully symmetrical, and the cortical regions for angles that closely match the location of the peripheral-right stimulus (phase angle=π/4) are mapped into only a small region (i.e., they are likely to represent lower SNR data). Another possibility is that the location of the activation to the peripheral right stimulus is more lateral than that of the peripheral left, which would mean it may not be entirely covered by the FOV of the array. Note that in Fig. 11(a), the locations of the active regions (contours at 75% of maximum ΔHbO) for the peripheral right stimulus are the outermost of all the conditions.

Figure 11(a) shows contour maps of activation at 75% of the maximum for all sessions. This figure shows that the average difference between the left central and peripheral centroids was $\Delta z_{\mathrm{AB}}=-9\pm 2\;\mathrm{mm}$ and the corresponding difference for the right centroids is $\Delta z_{\mathrm{CD}}=-9\pm 6\;\mathrm{mm}$, which are comparable with the value reported at the group level previously using a fiber-based HD-DOT of −4±7  mm for the right stimuli.^7^ The horizontal difference between the activations was $\Delta x_{\mathrm{AC}}=44\pm 5\;\mathrm{mm}$ which is again close to those ranges reported in the same publication ($\Delta x_{\mathrm{AC}}=37\pm 4\;\mathrm{mm}$). The vertical difference is −10±3  mm which is larger than the equivalent feature reported previously ($\Delta z_{\mathrm{AC}}=1\pm 5$), however, this is likely due to the inherent subject variability. The eccentricity experiment allowed us to demonstrate the consistency of the activation maps across multiple sessions. Furthermore, Fig 11(b) indicates that the regions of activation and the location of the maximum change are clearly consistent within the condition and distinguishable from one condition to another.

Figures 12(a) and 12(b) show the HRFs across all sessions for the central-left condition. The amplitude and morphology of the responses are remarkably consistent across different sessions, which occurred over the span of several days and weeks. This is confirmed by the HbO and HbR HRF temporal correlation matrices shown in Figs. 12(c) and 12(d), where the lowest correlation between the responses for any two sessions was 0.65. Figures 12(e)–12(h) show the spatiotemporal correlation matrices across all sessions and eccentricity mapping conditions. Note that the correlation values are high across all conditions except the peripheral right condition. This result was not unexpected since the response to this condition was of a lower amplitude and is thus more susceptible to noise. Figure 10(d) (lower panel) shows that this condition has the highest temporal variability and Fig. 10(p) indicates that the overlap for this condition is also the lowest. However, in general, the overlap and correlation maps of Figs. 11 and 12 clearly demonstrate that the observed activations are highly consistent in time and space across all 15 sessions. This is particularly remarkable when considering the fact that the cap was removed and reapplied for each session, and that the sessions occurred over a period of weeks. Comparing this result to test–retest examinations of traditional fNIRS systems,^59^^,^^62^ which generally show poorer repeatability, these results support the hypothesis that HD-DOT devices provide superior repeatability than fNIRS, this is likely due to the high amplitude and spatially continuous distributions. The spatiotemporal correlations for ΔHbO, obtained by concatenating the time series of the responses at all nodes across any two given sessions, show remarkably high correlation values (typically around R∼0.7), only the peripheral-right stimulus responses show lower values. The equivalent values for ΔHbR also demonstrate clear correlations, but lower values (typically R∼0.4) than ΔHbO due to the smaller contrast of the ΔHbR response.

### 4.4 Sparse Array fNIRS and HD-DOT Comparisons

To complement the results presented in this work and to explicitly examine the differences in performance that relate to high-density sampling, we included results and reconstructions from a simulated sparse array, shown in Fig. 13, which was obtained from a subset of the available high-density channels. We followed the same processing pipeline as with the HD array, but the SS channel regression was performed globally using the average of two SS channels in each hemisphere.^63^^,^^64^ In summary, our results show that when compared with sparse measurements, HD-DOT provides remarkably improved SNR, greater spatial specificity, and greater consistency across sessions. Figures 13(a)–13(d) show larger error bands associated with the hemodynamic responses for sparse measurements for all conditions. These time series emphasize the problem of low SNR with a sparse array, which is particularly evident for HbR responses and the central-right stimulus. In contrast, the temporal changes obtained with the high-density array show a much clear distinction between the rest and activation periods [Figs. 10(a)–10(d)]. On average, the SNR associated with HD-DOT derived HbO responses was approximately double (1.98 times) that of the sparse array. For HbR, the value was 1.67 times. Comparing Figs. 10(e)–10(l) and Figs. 13(e)–13(l) shows that a sparse array yields noisier reconstructions, often with foci that are offset by several centimeters relative to those obtained with HD-DOT. The sparse array also yields multiple activation foci in three out of four conditions. Finally, Figs. 13(m)–13(t) illustrate that the location of the response to each condition is less consistent across sessions for sparse measurements, particularly when the physiological signal is of a lower contrast (i.e., for HbR and the peripheral right condition). Taken together, these results demonstrate the dramatic improvements in spatial accuracy, robustness, and repeatability that can now be readily achieved with an HD-DOT approach.

### 4.5 Future Directions

In this work, we have introduced a new, wearable HD-DOT system with performance capabilities comparable to what were previously state-of-the-art, fiber-based HD-DOT devices. We employed a 12-module device, which was sufficient to cover the full adult visual cortex. However, the system is designed to allow expansion toward whole-head coverage in the adult. This development would permit high-density, full FOV DOT sampling of the human cortex for the first time. Such a technology would enable a huge expansion of possible DOT applications and would enable a standardization of measurement and analysis approaches across the field, as it would spell the end of array designs that vary with research lab, paradigm, and population.

The fiberless, light-weight, and wearable nature of the system demonstrated here offers great versatility for experimental application. It potentially supports prolonged recordings and crucially will permit unrestricted brain imaging in almost any environment. It should therefore provide a new tool to investigate cognitive function in ecologically valid settings. It is also important to mention the new possibilities offered by this system to study the infant brain; a population that has not benefited from the improvements offered by HD-DOT as much as others, largely due to physical constraints imposed by existing research devices.

Our current efforts are focused on enabling and validating these (and similar) technologies in other experimental circumstances, including outside of the laboratory. Combining this technology with eye-tracking and EEG holds particular promise, as it would potentially enable the acquisition of unprecedented levels of information about the brain as participants move, navigate, interact, and communicate in everyday environments, potentially encompassing the full human lifespan. Given the developments demonstrated here, we believe the stage is now set for wearable HD-DOT to become a standard neuroimaging tool across the neuroscience domain.
